# A Database Tool Integrating Genomic and Pharmacologic Data from Adrenocortical Carcinoma Cell Lines, PDX, and Patient Samples

**DOI:** 10.1158/2767-9764.CRC-24-0100

**Published:** 2024-09-11

**Authors:** Yasuhiro Arakawa, Fathi Elloumi, Sudhir Varma, Prashant Khandagale, Ukhyun Jo, Suresh Kumar, Nitin Roper, William C. Reinhold, Robert W. Robey, Naoko Takebe, Michael M. Gottesman, Craig J. Thomas, Valentina Boeva, Alfredo Berruti, Andrea Abate, Mariangela Tamburello, Sandra Sigala, Constanze Hantel, Isabel Weigand, Margaret E. Wierman, Katja Kiseljak-Vassiliades, Jaydira Del Rivero, Yves Pommier

**Affiliations:** 1 Developmental Therapeutics Branch, Center for Cancer Research, National Cancer Institute, National Institutes of Health, Bethesda, Maryland.; 2 Department of Medicine-Endocrinology/Metabolism/Diabetes, University of Colorado, Anschutz Medical Campus, Aurora, Colorado.; 3 Laboratory of Cell Biology, Center for Cancer Research, National Cancer Institute, National Institutes of Health, Bethesda, Maryland.; 4 National Institutes of Health, Rockville, Maryland.; 5 Department of Computer Science, Institute for Machine Learning, ETH Zurich, Zurich, Switzerland.; 6 Department of Medical and Surgical Specialties, Radiological Sciences, and Public Health, Medical Oncology Unit, University of Brescia, Azienda Socio Sanitaria Territoriale (ASST) Spedali Civili, Brescia, Italy.; 7 Section of Pharmacology, Department of Molecular and Translational Medicine, University of Brescia, Brescia, Italy.; 8 Department of Endocrinology, Diabetology and Clinical Nutrition, University Hospital Zurich, and University of Zurich, Zürich, Switzerland.; 9 Medizinische Klinik und Poliklinik III, University Hospital Carl Gustav Carus Dresden, Dresden, Germany.; 10 Division of Endocrinology and Diabetology, Department of Internal Medicine I, University Hospital, University of Würzburg, Würzburg, Germany.

## Abstract

Adrenocortical carcinoma (ACC) is a rare and highly heterogeneous disease with a notably poor prognosis due to significant challenges in diagnosis and treatment. Emphasizing on the importance of precision medicine, there is an increasing need for comprehensive genomic resources alongside well-developed experimental models to devise personalized therapeutic strategies. We present ACC_CellMinerCDB, a substantive genomic and drug sensitivity database (available at https://discover.nci.nih.gov/acc_cellminercdb) comprising ACC cell lines, patient-derived xenografts, surgical samples, and responses to more than 2,400 drugs examined by the NCI and National Center for Advancing Translational Sciences. This database exposes shared genomic pathways among ACC cell lines and surgical samples, thus authenticating the cell lines as research models. It also allows exploration of pertinent treatment markers such as MDR-1, SOAT1, MGMT, MMR, and SLFN11 and introduces the potential to repurpose agents like temozolomide for ACC therapy. ACC_CellMinerCDB provides the foundation for exploring larger preclinical ACC models.

**Significance:**

ACC_CellMinerCDB, a comprehensive database of cell lines, patient-derived xenografts, surgical samples, and drug responses, reveals shared genomic pathways and treatment-relevant markers in ACC. This resource offers insights into potential therapeutic targets and the opportunity to repurpose existing drugs for ACC therapy.

## Introduction

Adrenocortical carcinoma (ACC) is a rare malignancy affecting 1.5 to 2 million people per year. It has a dismal prognosis, with an overall 5-year combined survival of around 50% for all stages and an average survival of 14.5 months from the time of diagnosis (1, 2). ACC is a challenging disease with a broad range of clinical presentations, often presenting in an advanced stage with a large, locally invasive primary tumor or with Cushing syndrome, and poor prognosis with a 5-year mortality rate of 75% to 90%. The treatment of choice for localized primary or recurrent tumors is radical surgery. However, patients with metastatic or recurrent disease are infrequently curable by surgery alone, and even patients without objective and biochemical evidence of residual tumor after surgery often relapse (3). Chemotherapy offers limited benefit, although platinum-based therapies produce transient response rates of 25% to 30% (4). Currently, there is only one FDA-approved agent, mitotane (an analog of the insecticide dichlorodiphenyltrichloroethane) being used for the treatment of advanced ACC since the 1960s despite limited efficacy and significant toxicity (3).

ACC is highly heterogeneous. Genes involved in steroidogenesis are variably expressed, and their expression is driven by the SF-1 transcription factor encoded by the *NR5A1* gene (5). Activation of the Wnt signaling pathway has been observed in ACCs, often involving the dysregulation of β-catenin (6, 7). Overexpression of the insulin-like growth factor 2 gene (*IGF2*) occurs in approximately 90% of ACCs, and the interaction of IGF2 with the IGF-1 receptor activates the MAPK and PI3/Akt pathways, promoting adrenocortical proliferation (8). Additionally, mutations in the *TP53* tumor suppressor gene are frequent in patients with sporadic ACC, suggesting cell-cycle deregulation in ACC development (9). Common chromosomal abnormalities include loss of heterozygosity of 11p15 (seen in 93% of patients with ACC), gains in 1q, 5p, 5q, 6p, 6q, 8p, 8q, 9q, 10p, 11q, 12q, 13q, 14q, 15q, 16, 18q, 19, and 20q, and losses in 2q, 3, 4, 9p, 11, 13q, 18, 20p, and Xq (10). Approximately 10% of ACC cases are associated with hereditary cancer syndromes, including Li Fraumeni syndrome, multiple endocrine neoplasia type 1, Lynch syndrome (hereditary nonpolyposis colorectal cancer), Beckwith–Wiedemann syndrome, and familial adenomatous polyposis (refs. 11–13). In addition, ACCs have been reported in four patients with neurofibromatosis type 1 (NF1; refs. 14, 15) and in four patients with succinate dehydrogenase pathogenic mutations (16). Despite the recent insights into molecular mechanisms underlying ACC, no novel targeted therapies have been successful to date.

Current preclinical research models for ACC are limited. For decades, the only ACC cell lines available were SW-13 and H295, the latter being notable for its sustained steroid secretion even after decades of culture (17–19). There is considerable debate as to whether SW-13 is in fact of adrenocortical origin. SW-13 does not produce steroids, and it may have been a small cell lung cancer metastasis to the adrenal gland (19, 20). Only a few additional ACC lines have been reported in the last few years, including the CU-ACC1, CU-ACC2, MUC-1 [with companion patient-derived xenograft (PDX) models], TVBF-7, and JIL-2266 cell lines (18, 21–23).

The paucity of robust preclinical models and the rarity of ACCs have hampered therapeutic breakthrough and limited our comprehension of ACC’s underlying pathophysiology. To increase our understanding of genomics and potential therapeutic vulnerabilities, we performed integrated genomic and drug–response analyses of the four available ACC cell lines. We compared their genomics with the corresponding PDXs, as well as with six patients’ biopsy data. We also included data for the MUC-1, TVBF-7, and JIL-2266 cell lines in our genomic analyses (18, 21–23). The data are presented in this report and can be further queried with a novel open-access web-based application enabling anyone to mine the genomics and drug response of ACC cell lines, PDXs, and patient surgical samples. In this report, we present examples of representative molecular, pharmacologic, and genomic features [RNA sequencing (RNA-seq), methylome, and exome sequencing (Exome-seq)] and characteristics of ACC preclinical models compared with patient surgical resection samples.

## Materials and Methods

### Cell culture and reagents

The cell lines NCI-H295R and SW-13 were purchased from ATCC. CU-ACC1 and CU-ACC2 were provided by Drs. K. Kiseljak-Vassiliades and M. Wierman (18), MUC-1 cells by Dr. Hantel (21), and TVBF-7 cells by Dr. Sigala and Dr. Berruti (24). NCI-H295R cells were grown in 1:1 DMEM:F12 Nutrient Mixture (Thermo Fisher Scientific) supplemented with 2.5% Nu-Serum (Corning), 1% insulin, transferrin, and selenium supplement (R&D Systems), and 1% penicillin–streptomycin (Gibco). SW-13 cells were grown in DMEM (Thermo Fisher Scientific) supplemented with 10% FBS (GeminiBio) and 1% penicillin–streptomycin. CU-ACC1 and CU-ACC2 ACC cell lines were grown in F medium (18). MUC-1 cells were grown as described in a previous study (25). TVBF-7 cells were grown in DMEM–F12 supplemented with 10% FBS, 1% penicillin–streptomycin, amphotericin B (2.5 μg/mL), and 2 mmol/L glutamine. JIL-2266 cells were grown as described previously (22). All cell lines were cultured at 37°C with 5% CO_2_. They were tested negative for *Mycoplasma* using MycoAlert Mycoplasma Detection Kit (Lonza Bioscience) or qRT-PCR and authenticated by short tandem repeat analysis.

### ACC surgical samples

This study was conducted in accordance with the Declaration of Helsinki, and the research protocol was approved by the Institutional Review Board of the NCI. Written informed consent for Institutional Review Board–approved research at the NCI to collect and analyze surgical specimens was obtained from five ACC patients. Parts of six surgical specimens (one patient underwent two surgeries) were immediately kept on ice. After specimens were dissociated physically and enzymatically with collagenase, portions were stored at −80°C. Clinical information for the surgical samples is summarized in Supplementary Table S1.

### New, independent RNA-seq and DNA methylation analyses performed at the NCI

Total RNA was extracted from the four cell lines NCI-H295R, SW-13, CU-ACC1, and CU-ACC2 grown at the NCI and from six surgical tissues collected at the NCI using RNeasy Mini Kit (Qiagen). The RNA library was prepared using NEBNext Poly(A) mRNA Magnetic Isolation Module (NEB #E7490) _ UltraDirectional II (NEB #E7760) with NEB E6440 kits from New England Biolabs. Sequencing was carried out in the Illumina NextSeq 550 instrument with NextSeq 500/550 Mid Output Kit v2.5 (150 cycles) with 75 × 75 pair end configuration.

For the samples processed at the NCI, DNA was extracted using DNeasy Blood & Tissue Kit (Qiagen). The extracted quantity was measured, and quality control was performed using a NanoDrop One spectrophotometer (Thermo Fisher Scientific). Comprehensive analysis of DNA methylation sites was conducted using the Illumina 850 K Epic Methylation array (Illumina).

### University of Colorado genomics data

We evaluated the RNA-seq and Exome-seq raw data (fastq files) for the CU-ACC1, CU-ACC2, and NCI-H295R cell lines and for the two corresponding PDXs. Patient information on the original tumors and the methods used to establish the PDXs and cell lines have been previously described (18, 23).

### Data processing

All NCI and University of Colorado RNA-seq and Exome-seq raw data were processed uniformly using the NCI CCBR pipelines (https://github.com/CCBR/RNA-seek and https://github.com/CCBR/exome-seek) to produce normalized gene expression and mutation calls. Gene level mutations, gene promoter and gene body methylation, and gene copy numbers were generated as previously described (26, 27).

### University of Zurich WGS data

Whole-genome sequencing (WGS) data from NCI-H295R and MUC-1 cells were analyzed according to methods previously presented (24). WGS data processing for TVBF-7 was performed by BGI Genomics, Inc., including data filtering (removal of adapters, contamination, and low-quality reads from raw reads), alignment of reads to the human reference genome (University of California Santa Cruz build HG19) using the Burrows-Wheeler Aligner software, sequence quality assessment, sequence depth distribution, coverage uniformity assessment, and variant calling analysis [base quality score recalibration and Genomic Variant Call Format (tool: GATK)] were performed by applying WGS standard bioinformatics.

### Drug cytotoxicity data

For the data generated in our laboratory, NCI-H295R, SW-13, CU-ACC1, and CU-ACC2 cells were plated on 384-well white plates at a density of 300 cells/well; after 24 hours of incubation, the indicated drugs were added, and cells were incubated for 72 hours. Cell viability was evaluated using CellTiter-Glo (Promega) with SpectraMax i3x (Molecular Devices) according to the manufacturer's instructions. Drugs were obtained from the Developmental Therapeutics Program (NCI).

For the data generated at the National Center for Advancing Translational Sciences (NCATS), NCI-H295R, CU-ACC1, and CU-ACC2 cells were seeded into 1,536-well tissue culture–treated plates at a density of 500 to 1,000 cells/well in 5 μL of growth medium using a Multidrop Combi dispenser (Thermo Fisher Scientific). After cell addition, 23 nL of mechanism interrogation plate 5.0 compound (28, 29) was added to individual wells (11 concentrations were administered for all compounds in separate wells). Three μL of CellTiter-Glo (Promega) was loaded to each well, and the plate was covered with a stainless steel lid and incubated at room temperature for 15 minutes. Luminescence was read using a ViewLux microplate imager (PerkinElmer). Dose–response curves for compounds were normalized against DMSO and empty well controls for each plate. All single-drug screening data are available both at the NCATS_CellMinerCDB and ACC_CellMinerCDB websites (https://discover.nci.nih.gov/; ref. 27).

### Western blot analysis

Cells were lysed in RIPA buffer (150 mmol/L NaCl, 50 mmol/L Tris-HCl (pH 7.5), 1 mmol/L EDTA, 1% NP40, 0.1% SDS, and 0.5% sodium deoxycholate) containing protease inhibitor cocktail (Cell Signaling Technology) and phosphatase inhibitor (Thermo Fisher Scientific). Cell lysates were loaded into wells of Novex Tris-Glycine gels (Invitrogen), electrophoresed, and transferred to Immun-Blot PVDF membranes (Bio-Rad). The membranes were incubated with primary Abs [MGMT (58121, Cell Signaling Technology), MDR-1 (C219, from Dr. Robert W Robby at the NCI), BCRP (BXP-21, also from Dr. Robert W Robby), TOP1 (sc-10783, Santa Cruz Biotechnology), SLFN11 (sc-515071, Santa Cruz Biotechnology), and GAPDH (GTX100118, GeneTex)] overnight in PBS-T buffer at 4°C, followed by incubation with horseradish peroxidase–labeled secondary Abs (Cell Signaling Technology). The membranes were developed with SuperSignal West Pico Plus or Femto Substrate (Thermo Fisher Scientific) according to the manufacturer’s instructions and imaged with the ChemiDoc imaging system (Bio-Rad). Band intensities were quantified using ImageJ software (RRID: SCR_003070).

### Immunofluorescence microscopy

The cells were grown on coverslips and were fixed with 4% paraformaldehyde for 10 minutes, followed by incubation for 30 minutes in BSA blocking buffer. Primary Abs for TRF2 (NB110-57130, Novus Biologicals) and PML (sc-966, Santa Cruz Biotechnology) in the blocking buffer were incubated for 1 hour. Coverslips were then washed in PBS three times and incubated with secondary Abs (Alexa 488, A11034 and Alexa 568, A11031 from Thermo Fisher Scientific) in the blocking buffer for 30 minutes. The cells were washed three times in PBS and were mounted on the coverglass using VECTASHIELD Antifade Mounting Medium with 4',6-diamidino-2-phenylindole (DAPI). The images were captured using a Zeiss LSM 780 confocal microscope.

### Data availability

The data presented in the figure are publicly available and retrievable on the ACC_CellMinerCDB website (https://discover.nci.nih.gov/acc_cellminercdb). Any additional information needed to reanalyze the data reported in this article is available from the corresponding author upon reasonable request.

## Results

### Overview of ACC CellMiner

**Figure 1:**
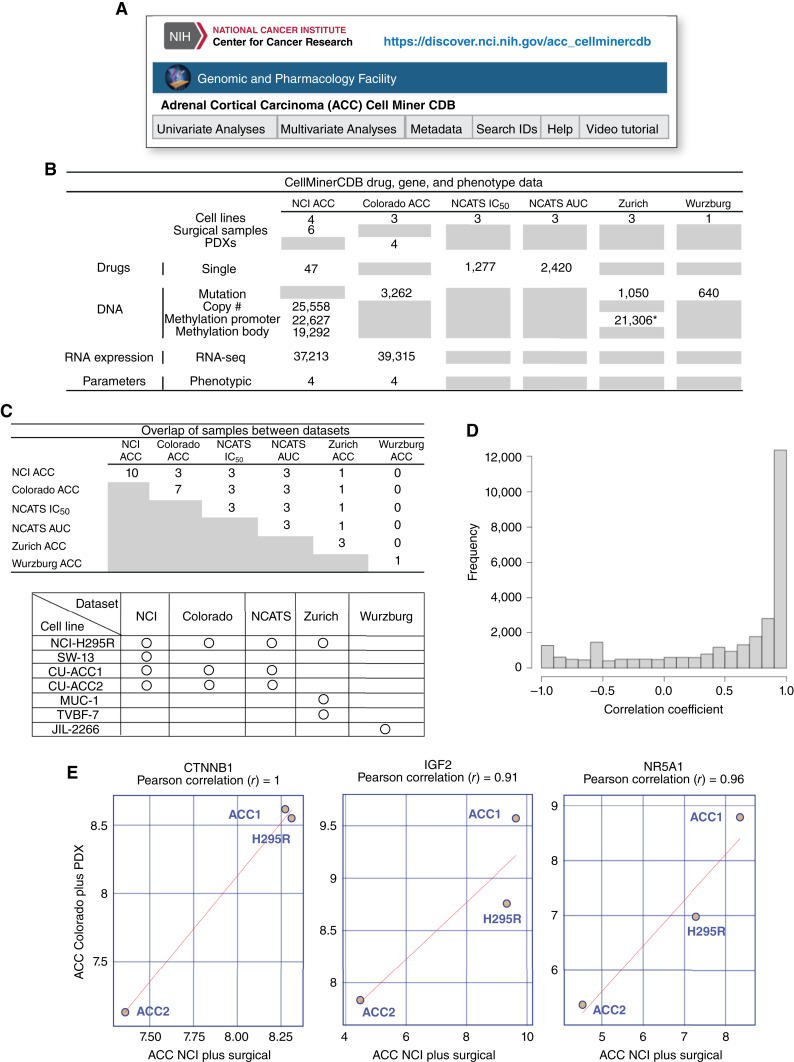
Overview of the datasets and reproducibility of ACC CellMiner. **A,** URL and snapshot of the website for ACC_CellMinerCDB. **B,** Summary of the molecular and drug activity data for cell lines, patient-derived mouse xenografts, and surgical samples included in ACC CellMiner. For each type of molecular and drug data, numbers indicate how many genes or drugs are included. Gray boxes indicate items with no data. **C,** Table of samples overlapping between datasets (top) and cell lines included in each dataset (bottom). **D,** Distribution of gene expression correlation between “ACC NCI plus surgical” and “ACC Colorado plus PDX” data sets. **E,** CTNNB1, IGF2, and NR5A1 gene expressions in the two data sets are plotted, and Pearson’s correlation coefficients are shown at the top of the plots. *Only the TVBF-7 cell line has methylation data.

We developed the web application ACC CellMiner (https://discover.nci.nih.gov/acc_cellminercdb) upon the architecture of our existing CellMinerCDB web tools (https://discover.nci.nih.gov/; Fig. 1A; ref. 26). This application compiles and integrates genomics data from various sources, including the University of Colorado, the NCI Center for Cancer Research (NCI-CCR), the University of Zurich, and the University Hospital of Wurzburg. Additionally, ACC_CellMiner incorporates drug–response data obtained from the NCI and the NCATS. The omics and drug activity data from the ACC cell lines, PDXs, and surgical samples are summarized in Fig. 1B. Samples that overlap between data sets and the cell lines included in each dataset are summarized in Fig. 1C. The data for the cell lines CU-ACC1, CU-ACC2, and NCI-H295R overlap the “NCI ACC plus surgical”, the “ACC Colorado plus PDX,” and the NCATS datasets. The former contains data on ACC cell lines and surgical samples, whereas the University of Colorado dataset (CU) contains data on ACC cell lines and the PDXs from which the (CU-ACC) cell lines were derived.

### NCI-CCR versus CU cell line comparisons

As whole-genome gene expression is included in ACC CellMiner for the NCI-H295R, CU-ACC1, and CU-ACC2 cell lines in both the dataset from the University of Colorado (CU; ref. 18) and the NCI-CCR, we examined the correlations of gene expression between the two datasets. For the majority of genes (more than 12,000), the correlation coefficients between the datasets were greater than 0.9 (Fig. 1D). As examples, gene expression correlations for genes highly expressed in ACCs, such as *CTNNB1*, *IGF2*, and *NR5A1*, are shown in Fig. 1E. These comparisons exemplify the reproducibility of the RNA-seq data tested independently and the stability of the cell lines.

### Gene expression analyses

**Figure 2:**
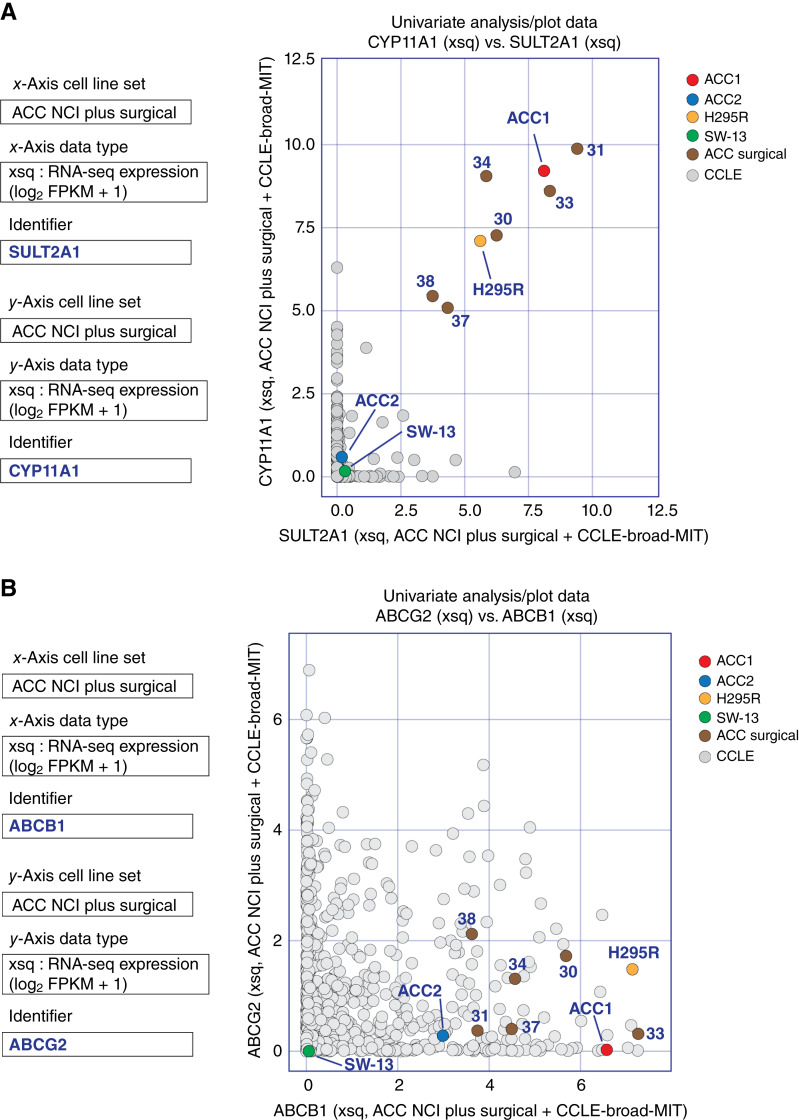
RNA-seq (xsq) expression of hormonal and drug efflux genes in the ACC cell lines and surgical samples. **A,** Univariate analysis scatterplot of the hormonal genes SULT2A1 (sulfotransferase family 1A member 1) vs. CYP11A1 (cytochrome P450 family 11 subfamily A member 1) transcript levels in ACC NCI cell lines and surgical samples. Transcript levels of the two genes in the 1,011 cell lines in the CCLE dataset were merged. **B,** Univariate analysis scatterplot of the drug transporters ABCB1 (MDR1) vs. ABCG2 (BCRP) transcript expression levels in ACC NCI cell lines and surgical samples. Transcriptional expression levels of ABCB1 and ABCG2 in the 1,011 cell lines of the CCLE dataset were merged with the ACC data.

More than half of ACC cases are hormone producing (30–32). Because steroidogenesis in ACC is not only a molecular marker of cancer cell differentiation and characterization but also a poor clinical prognostic factor (7, 31), we looked at the expression of genes involved in steroid metabolism in the cell lines and surgical sample datasets (NCI ACC plus surgical; see Fig. 1B) and compared the expression of hormonal genes in the ACC cell lines and the cell lines in the Cancer Cell Line Encyclopedia (CCLE) dataset, which does not include ACC. Figure 2A shows that the steroid-producing ACC cell lines (NCI-H295R and CU-ACC1) and the surgical samples from the NCI express both *CYP11A1* (encoding cytochrome 11A1, a cytochrome P450 that catalyzes the synthesis of pregnenolone from cholesterol) and *SULT2A1* (encoding a sulfotransferase that catalyzes the sulfonation of steroids). In contrast, CU-ACC2, a non–steroid-producing ACC cell line (18) and SW-13, a small cell carcinoma cell line, exhibit no expression of *CYP11A1* or *SULT2A1*. Figure 2A shows that only a few of the 1,011 cancer cell lines of the CCLE dataset expressed significant levels of *SULT2A1* and *CYP11A1* (e.g., colorectal cancer cell lines such as OUMS-23 and CACO2, as well as hepatocellular carcinoma cell line HuH-7).

Drug efflux transporters are major contributors to the resistance of cancers to chemotherapy (33). ACCs are known to overexpress the *ABCB1* gene, which encodes the ABC transporter MDR1, and this is likely one of the reasons why conventional anticancer drugs are ineffective in ACC (34). To test whether ACC cell lines overexpress drug efflux transporters, the gene expression of *ABCB1* and *ABCG2* (encoding MDR1 and BCRP, respectively) were analyzed. High expression of *ABCB1* was observed in the ACC cell lines and the six surgical samples, as well as in many of the cancer cells lines of the CCLE. NCI-H295R and CU-ACC1 showed higher expressions of *ABCB1* than most of the cell lines in the CCLE dataset (Fig. 2B). In contrast, SW-13 showed no expression of *ABCB1*. Consistent with the overexpression of *ABCB1* transcripts, Western blot analyses showed overexpression of *ABCB1* in CU-ACC1 and NCI-H295R (35). Moreover, the activity of docetaxel, a known substrate of MDR1 (33), was inversely correlated with *ABCB1* expression (Supplementary Fig. S1).

### Comparison of gene expression in the ACC cell lines and their corresponding PDXs

**Figure 3:**
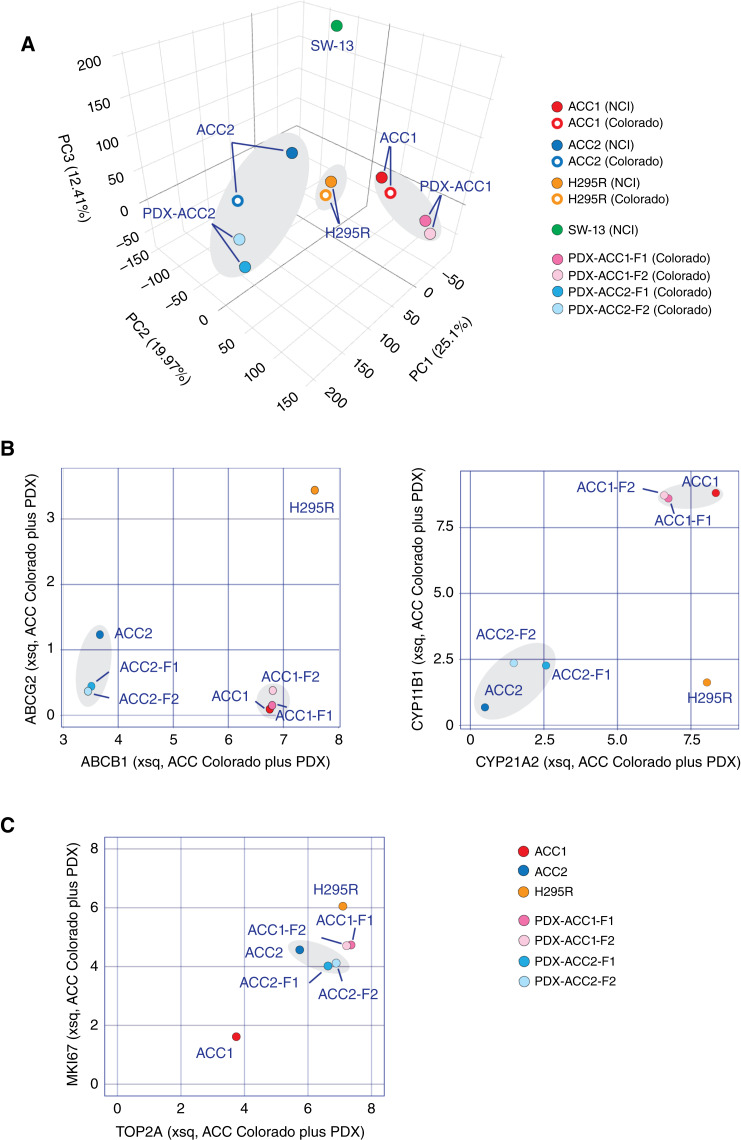
Comparison of gene expression of CU-ACC cell lines and original PDXs. **A,** Overall correlation between gene expression in the cancer cell lines evaluated at the NCI and CU and their corresponding PDXs. Areas shaded in gray highlight the grouping of corresponding ACC cell lines and PDXs. **B,** Examples of coherent genes. Univariate analysis scatterplot of ABCB1 (MDR1) transcriptional expression level vs. ABCG2 (BCRP) and CYP21A2 transcriptional expression level vs. CYP11B1 transcriptional expression level. **C,** Example of differentially expressed genes related to cellular proliferation for the CU-ACC1 and CU-ACC2 cell lines and corresponding PDXs.

To compare the ACC cell lines and their corresponding PDXs, we performed principal component analysis of global gene expression for the ACC cell lines and PDXs. Figure 3A shows that gene expression in the CU-ACC1 and CU-ACC2 cell lines evaluated independently at the NCI and Colorado University (CU) and the parental PDXs grouped with each other. NCI-H295R in the NCI and CU datasets was also in proximity with the ACC1 and ACC2 clusters and distant from SW-13. This analysis shows the similarities between the ACC cell lines and the PDXs from which they were derived.

Examples of specific genes evaluated with ACC_CellMinerCDB are shown in Fig. 3B and C. The expression of *ABCB1* and *ABCG2* using the univariate analysis tool of ACC_CellMinerCDB shows that the CU-ACC1 cell line and its parental PDXs (PDX-ACC1-F1 and PDX-ACC1-F2) are grouped together as high-*ABCB1* expressers. Likewise, the CU-ACC2 cell line and its parental PDXs (PDX-ACC2-F1 and PDX-ACC2-F2) are closely grouped. Both are far from NCI-H295R, which expresses high levels of both *ABCB1* and *ABCG2*. Thus, the expression of ABC transporters (ABCB1 and ABCG2) remains largely unchanged during passaging of PDXs and the establishment of the cell lines from the PDXs.

Comparing the expression of genes encoding steroid-metabolizing enzymes (*CYP21A2* and *CYP11B1*) between the cell lines and the PDXs showed that the expression of those genes was also maintained during cell line establishment [Fig. 3B (right)]. However, a small number of genes seemed differently expressed in the cell lines and their corresponding PDXs. For instance, the CU-ACC2 cell line and the original PDXs were highly expressed and in proximity, whereas the CU-ACC1 cell line showed significantly lower expression than the original PDXs for *MKI67* and *TOP2A*, which are indicators of cell proliferation (Fig. 3C). We conclude that most genes exhibit comparable expression in the ACC cell lines and the PDXs from which they were derived.

### Genomic signatures and adrenocortical biomarkers in the ACC cell lines and surgical samples

Like the other CellMinerCDB websites (26), ACC_CellMinerCDB not only includes single gene expression analysis tools but also four molecular signatures: NE, APM, RepStress, and ADS. The NE (neuroendocrine) signature is based on the expression of 25 genes (36), the APM (antigen presentation machinery) score on 18 genes (37), and the RepStress (replication stress) signature on the transcript expression signature of 18 genes (38, 39).

**Figure 4:**
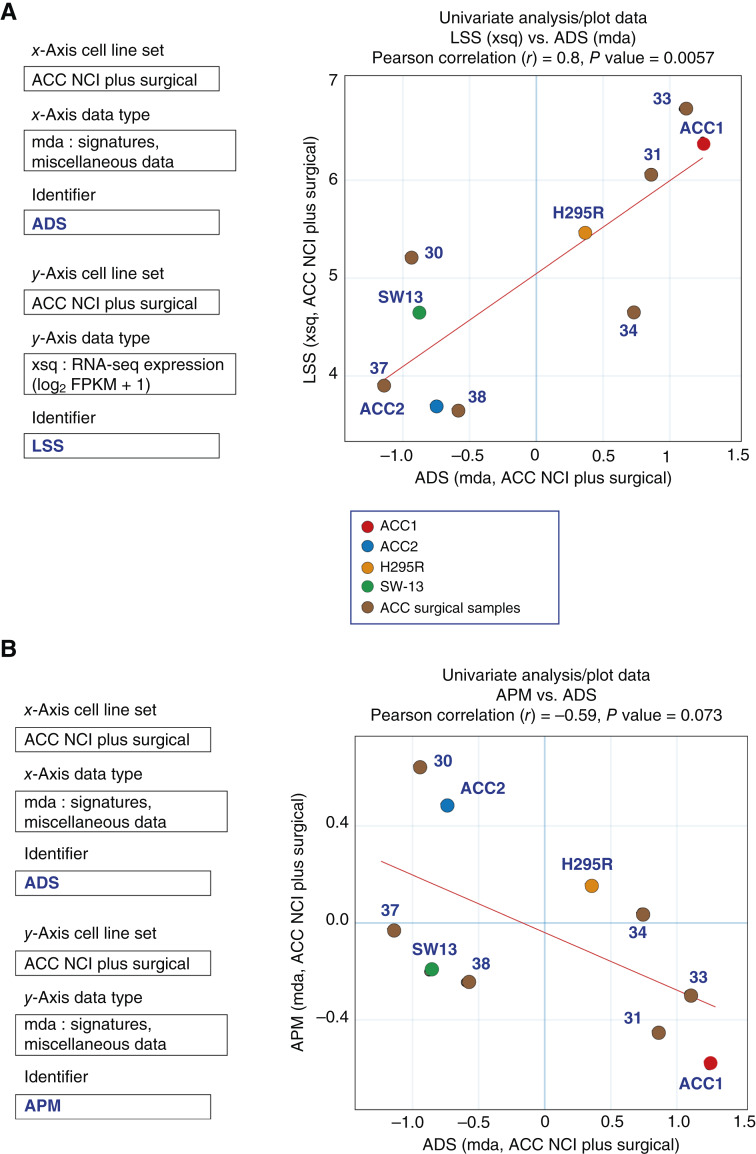
Examples of biomarker signatures in the ACC cell lines and surgical samples. **A,** The ADS varies across ACC cell lines and surgical samples and is correlated with the expression of *LSS* (lanosterol synthase), a key enzyme in cholesterol biosynthesis. **B,** The ADS is negatively correlated with the APM score.

Based on The Cancer Genome Atlas (TCGA) database, Zheng and colleagues (7) proposed the adrenocortical differentiation score (ADS). It is calculated from the expression of 25 genes, including steroid metabolism genes, cholesterol transporter genes, and their transcriptional regulator SF-1 (steroid factor 1 encoded by *NR5A1*), which are involved in adrenocortical differentiation and have been shown to affect the prognosis of ACC cases (5, 40). The ADS is included in ACC_CellMinerCDB as a gene signature (“mda: signatures, miscellaneous data”). As expected, analyses in the cell line and surgical sample datasets show that *NR5A1* gene expression levels correlate well with ADS (Pearson correlation *r* = 0.68, *P* value 0.03; Supplementary Fig. S2A). Furthermore, the correlation between *NR5A1* gene expression and ADS is even stronger when SW-13, the small cell cancer cell line, is excluded, suggesting the importance of NR5A1 as a master transcription factor in adrenocortical differentiation. Notably, another gene, *LSS*, encoding lanosterol synthase and which is not included in the ADS, shows highly significant correlation with ADS (Pearson correlation *r* = 0.8, *P* value 0.0057; Fig. 4A). These results demonstrate the applicability of the ADS to the ACC cell lines and surgical samples. They also show that samples and cell lines can be defined as high-steroid genotype (CU-ACC1 and surgical samples 31 and 33) and low-steroid genotype (CU-ACC2, SW-13, and surgical samples 30, 37, and 38; Fig. 4A).

The APM score, which is present in the “mda” tab of CellMinerCDB applications, reflects the potential sensitivity of cancer cells to immune checkpoint inhibitors (ICI) (26). The higher the score, the greater the expression of antigen-presenting genes. Figure 4B shows that ADS is negatively correlated with the APM score, which suggests that ACC cells with high steroid metabolism tend to be less visible to the innate immune responses.

### Telomere maintenance in the ACC samples

**Figure 5:**
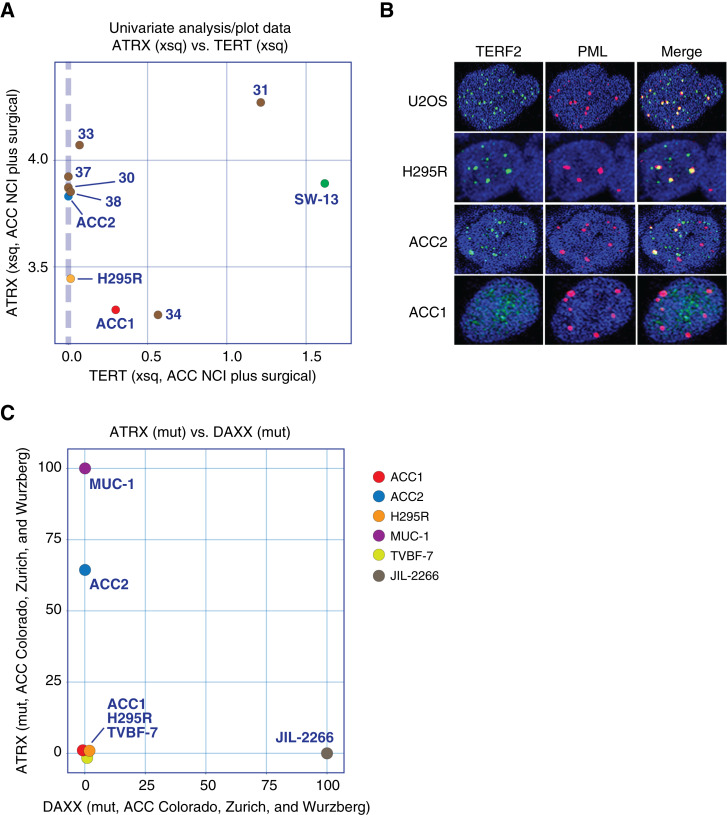
Several ACC cell lines and surgical samples do not express TERT. **A,** Univariate scatterplot of TERT transcriptional expression levels vs. ATRX transcriptional expression levels in the ACC cell line and surgical sample data set. **B,** Alternative lengthening of telomeres (ALT)-associated PML body formation. Representative confocal micrograph images of ALT cell line (U2OS) and ACC cell lines. Cells were fixed and immunofluorescently labeled with Abs against TERF2 and PML; TERF2 was stained green, PML red, and nuclei DAPI blue. **C,** DAXX and ATRX mutations in Colorado, Zurich, and Wurzburg data sets. Mutation scores in each dataset were collected and plotted; MUC-1 and CU-ACC2 have ATRX mutations, and JIL-2266 has DXAA mutation.

Because mutations in the promoter region of the *TERT* gene (encoding telomerase), which activate *TERT* transcription, have been reported in ACC, we checked the ACC cell line and surgical samples for *TERT* expression. Unexpectedly, we found that the cell lines NCI-H295R and CU-ACC2 and at least three surgical samples had no *TERT* mRNA expression (Fig. 5A). We further examined the colocalization of TERF2 (telomeric repeat–binding factor 2; TRF2) and PML (PML nuclear body scaffold), a hallmark of ALT (alternative lengthening of telomeres), a telomere-independent telomere maintenance mechanism, in the ACC cell lines. Colocalization was observed in NCI-H295R and CU-ACC2, suggesting that these cells have an ALT phenotype (Fig. 5B, U2OS is a positive control). No colocalization was observed in CU-ACC1. We also checked all ACC cell lines for *ATRX* (α-thalassemia/mental retardation syndrome X-linked gene) and *DAXX* (death domain–associated protein) mutations associated with the ALT phenotype and found *ATRX* mutations in CU-ACC2 and MUC-1 and *DAXX* mutations in JIL-2266 (Fig. 5C; Supplementary Table S2).

### DNA alterations in the ACC cell lines

Previous comprehensive genome sequencing studies have explored driver mutations in ACC clinical samples and identified abnormalities in the p53/Rb and the Wnt/β-catenin signaling pathways, suggesting that these pathways are critical for ACC pathogenesis (7, 41).

**Figure 6:**
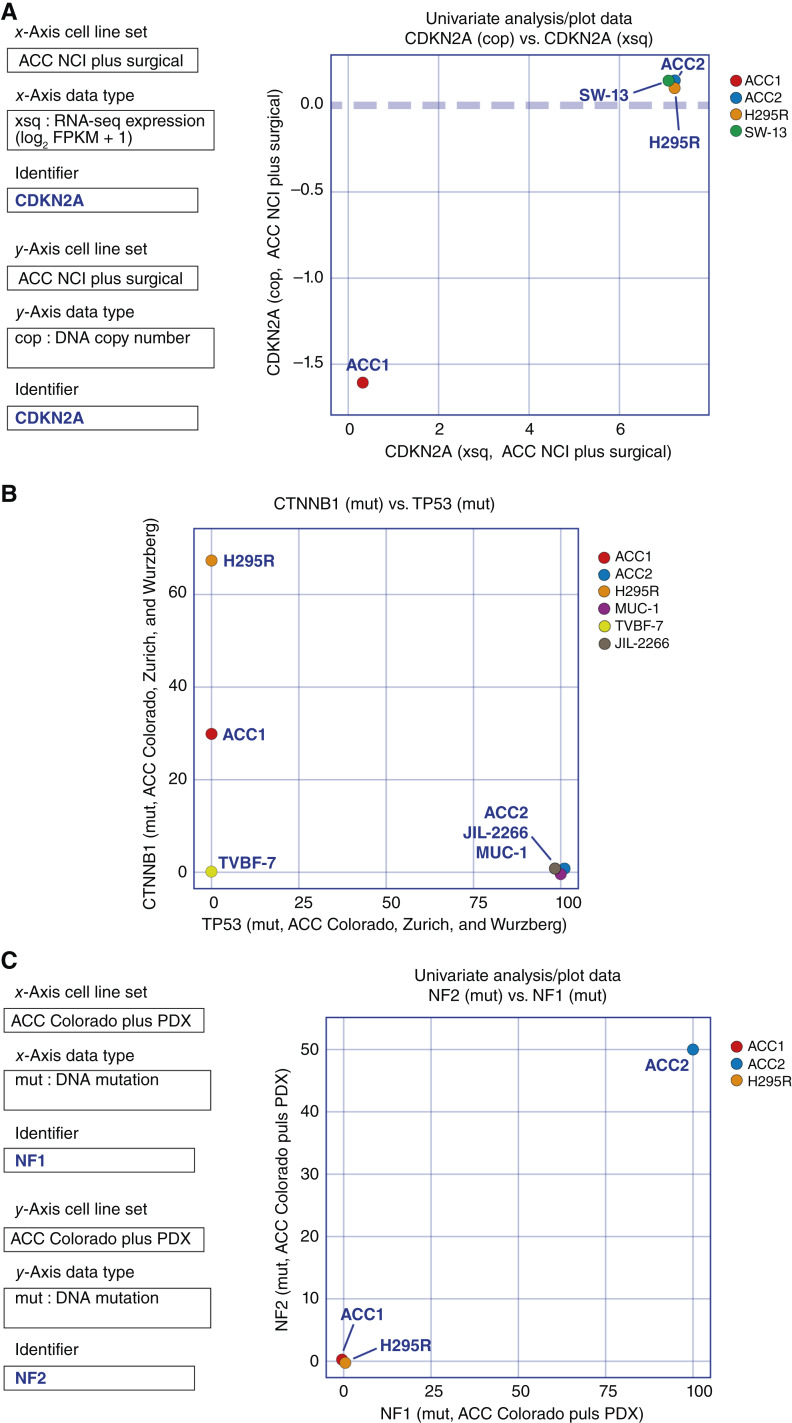
Examples of gene copy number variations and mutations in the ACC cell lines. **A,** CU-ACC1 is defective in CDKN2A. Univariate scatterplot of CDKN2A transcriptional expression levels vs. CDKN2A gene copy number in the ACC NCI cell line data set. **B,** Gene mutations in the ACC cell lines; mutation scores were collected from the Colorado, Zurich, and Wurzburg datasets and plotted; CU-ACC2, MUC-1 and JIL-2266 are homozygous TP53 mutants, CU-ACC1 and H595R exhibit heterozygous β-catenin (CTNNB1) mutations. **C,** CU-ACC2 harbor homozygous *NF1* gene mutation and heterozygous *NF2* gene mutation in the ACC NCI cell line data set.

CDKN2A, a key regulator of the p53/Rb signaling pathway, has also been reported to have recurrent loss-of-function mutations or gene defects in ACC (41). Accordingly, we found a deletion of the *CDKN2A* gene and lack of *CDKN2A* transcripts in CU-ACC1 cells (Fig. 6A). Previous reports on ACC cell lines have shown that CU-ACC2, MUC-1, and JIL-2266 have mutations in *TP53* and that CU-ACC1 and NCI-H295R harbor mutations in β-catenin (encoded by *CTNNB1*; refs. 18, 22, 24), and we confirmed these results as displayed in ACC_CellMinerCDB (Fig. 6B; Supplementary Table S2). The NCI-H295R cell line has been reported to have a homozygous deletion of exons 8 to 9 in the TP53 gene and a homozygous deletion of c.862_2787del1926 in the RB1 gene (42, 43). However, our copy number analysis using methylation arrays of the NCI dataset did not detect these deletions. In the NCI-H295R cells, the TP53 gene is located within a large region with a copy number of 2n. Methylation arrays typically have a limited number of probes in the gene body, which means that small-scale copy number changes could be missed. In the same analysis, the RB1 gene did not show a copy number value. This is because methylation probes with high-detection *P* values were removed, and many probes within the RB1 gene body exhibited such high *P* values.

ACC has been reported to occur in neurofibromatosis type 1 patients with pathologic germline mutations in *NF1* (14, 15). In addition, *NF1* is listed as one of the driver mutations for ACC in the TCGA report (7). In another comprehensive genome sequencing study, *NF2* mutations were also listed as a recurrent genetic abnormality for ACC (44). Exome sequencing detected a mutant allele in *NF1* that was frameshifted by a 7-base insertion in exon 30 within the Ras-GAP domain in CU-ACC2 (45). Additionally, one of the *NF2* alleles showed a detrimental missense mutation, demonstrating profound alterations of the NF1/2 pathways in CU-ACC2 cells (Fig. 6C).

### Anticancer drug sensitivity of the ACC cell lines

The activity of approximately 2,400 drugs in CU-ACC1, CU-ACC2, and NCI-H295R cells can be explored in ACC_CellMinerCDB both in the NCI-CCR and NCATS datasets, which we recently described and made openly accessible (see Fig. 1B; ref. 27).

**Figure 7:**
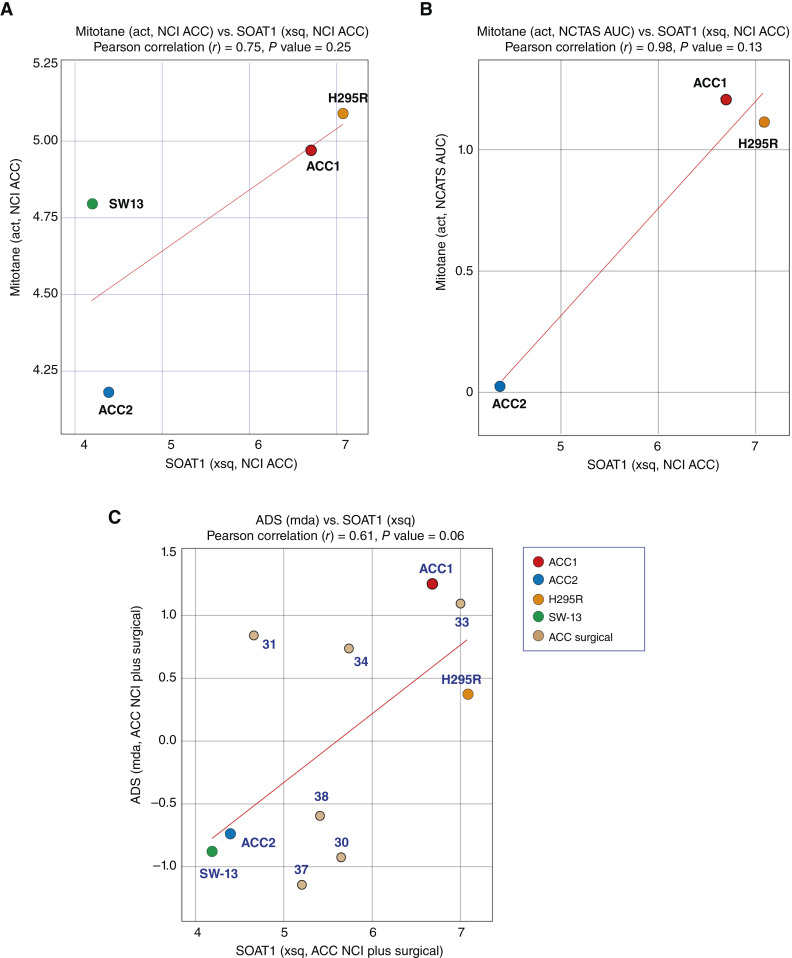
**A,** Correlation between the activity of mitotane tested at the NCI and the expression of SOAT1. **B,** Correlation between the activity of mitotane tested independently at the NCAT and the expression of SOAT1. **C,** Correlation between the expression of SOAT1 and the ADS signature.

Because the mainstay for treating ACC includes mitotane (3, 46), mitotane was included in our drug screening. It is also among the 2,400 drugs tested at the NCATS (see Fig. 1B; refs. 26, 27). Figure 7 shows that the activity of mitotane tested both in our laboratory (A) and within the NCATS screen (B) is correlated with the expression of its target, sterol-O-acyl transferase 1 (encoded by the *SOAT1* gene; ref. 47). Furthermore, consistent with the biological function of SOAT1 for steroid hormone biosynthesis, analyses with ACC_CellMinerCDB showed that the expression of *SOAT1* is correlated with high ADS values in the steroidogenic cell lines NCI-H295R and CU-ACC1 and the six surgical patient samples (Fig. 7C).

The standard first-line chemotherapy for advanced or relapsed ACC is EDP-M (etoposide, doxorubicin, cisplatin, and mitotane). However, there is no second-line chemotherapy established (48). In this context, the orally administered alkylating agent temozolomide is a potential candidate for second-line chemotherapy. Temozolomide alkylates guanine in DNA to produce O^6^-methylguanine, which forms a mismatch with thymine during DNA replication. The cell removes thymine through a mismatch repair (MMR) mechanism; however, as long as O^6^-methylguanine exists, the mismatch is formed again, and cell death is induced in a MMR-dependent manner as this futile cycle is repeated (49).

**Figure 8:**
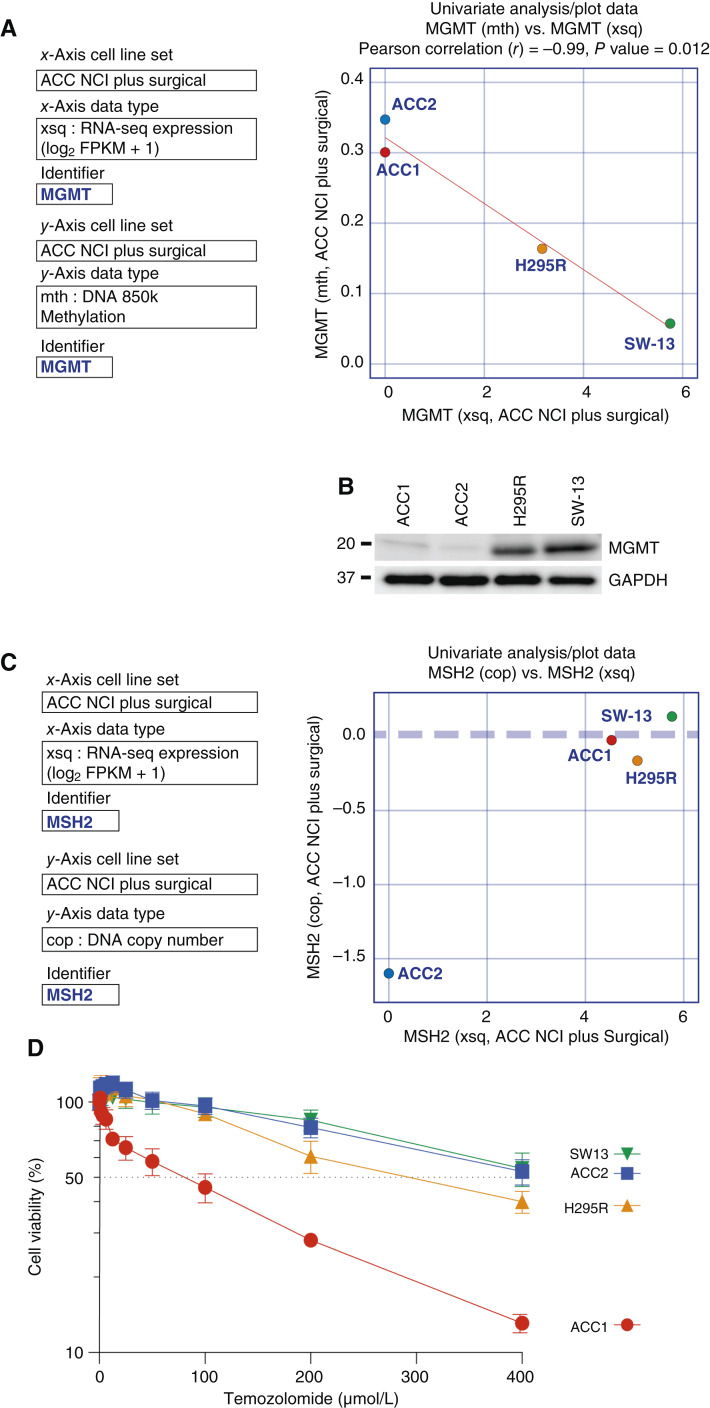
The CU-ACC1 cell line is sensitive to temozolomide as it lacks MGMT and is MMR proficient, whereas CU-ACC2 cells are resistant to temozolomide because of MMR deficiency. **A,** CU-ACC1 and CU-ACC2 do not express *MGMT* transcripts. Univariate scatterplot of *MGMT* transcriptional expression levels vs. *MGMT* gene promoter methylation levels in the ACC cell line dataset. **B,** MGMT protein expression levels in CU-ACC1, CU-ACC2, NCI-H295R, and SW-13. Proteins were extracted from each cell line, and MGMT expression was assessed by Western blotting. **C,** The CU-ACC2 cell line is defective in MMR due to lack of expression of the *MSH2* gene. Univariate scatter plot of *MHS2* transcript levels vs. *MSH2* gene copy number in the ACC cell lines data set. **D,** Dose–response curves of temozolomide in CU-ACC1, CU-ACC2, NCI-H295R, and SW-13. Cell viability was assessed after 72 hours under the indicated drug concentrations by CellTiter-Glo assay.

Because O^6^-methylguanine–DNA methyltransferase (MGMT) is known to cancel the effect of temozolomide by removing the methyl moiety from O^6^-methylguanine, we checked the expression of *MGMT* in the ACC cell lines (Fig. 8A and B). *MGMT* expression correlated negatively with its promoter methylation levels, and CU-ACC1 and CU-ACC2 did not show *MGMT* expression (Fig. 8A). Lack of the MGMT protein in CU-ACC1 and CU-ACC2 was confirmed by Western blotting (Fig. 8B).

The CU-ACC2 cell line, derived from a Lynch syndrome patient, had a heterozygous deletion of MSH2 exons 1 to 6 in the germ line (18). Loss of heterozygosity, deletion of the *MSH2* gene, and lack of MSH2 transcript expression in CU-ACC2 were readily detected by ACC CellMinerCDB (Fig. 8C). This deletion was consistently observed in the corresponding PDXs ACC2-F1 and ACC2-F2 (Supplementary Fig. S3A). The presence of an intact mismatch repair mechanism determines temozolomide sensitivity (49). Accordingly, we found that only CU-ACC1 cells are sensitive to temozolomide, whereas CU-ACC2 and NCI-H295R are resistant (Fig. 8D). This selective sensitivity was also demonstrated in the NCATS screening dataset (Supplementary Fig. S3B). These data indicate the potential of temozolomide as a second-line treatment for ACC (50) and the relevance of evaluating MGMT and MMR in ACC.

Because SLFN11 (Schlafen 11) expression in cancer cells has been reported to determine their sensitivity to a wide range of DNA-damaging anticancer drugs, including topoisomerase, PARP inhibitors, and platinum-based drugs (50), we examined SLFN11 expression in theACC cell lines and surgical samples. The ACC cell lines (NCI-H295R, CU-ACC1, and CU-ACC2) did not express SLFN11 (Supplementary Fig. S4A; ref. 35), and lack of SLFN11 expression was significantly correlated with *SLFN11* promoter methylation (Supplementary Fig. S4A). This finding is consistent with the fact that approximately 50% of cancer cell lines do not express SLFN11 (51), which otherwise would act to suppress cancer cell growth. The small cell carcinoma cell line SW-13 expresses SLFN11 both at the protein and transcription levels (Supplementary Fig. S4A; ref. 35) and, as expected (50), was found the most sensitive to topotecan among the four cell lines examined (Supplementary Fig. S4B). Additionally, *SLFN11* expression is known to be regulated by the transcription factor FLI1, which we found not to be expressed in the ACC cell lines (CU-ACC1, CU-ACC2, and NCI-H295R), and the expression levels of *SLFN11* in the ACC cell line and surgical samples correlated positively with the expression of *FLI1* (Supplementary Fig. S4C). Notably the expression of *SLFN11* in SW-13 cells is not related to *FLI1* (Supplementary Fig. S4C).

## Discussion

ACC_CellMinerCDB is the first and, to our knowledge, the only genomic database designed specifically for the exploration of ACC preclinical models (cell lines and PDXs) with the inclusion of six surgical ACC samples. ACC_CellMinerCDB is designed as a dynamic resource that can be expanded and integrated as new ACC cell lines and preclinical models become available to facilitate the development of personalized treatment strategies in the context of the rarity and heterogeneity of ACC, which remains a challenge for patients, researchers, and clinicians. In the era of precision medicine, the need for comprehensive genomic resources has never been greater, and there have been several reports of comprehensive genomic analyses for ACC patient samples, including the TCGA and ENS@T cohorts (6, 7, 41, 44, 52, 53). However, genomic analyses of preclinical model have been lacking. ACC_CellMinerCDB, with its extensive genomic and drug data and ease of operation, opens perspectives for in-depth studies of the genomic landscape and drug therapies of ACC.

In the past few years, new cell lines (CU-ACC cell lines, MUC-1, TVBF-7, and JIL-2266) as well as 3D culture models and PDXs have been reported, expanding the preclinical models available for ACC (22, 23, 54–56). A notable finding of our study is that ACC cell lines maintain features of typical ACC and retain their genomic characteristics over time, as observed when tested separately at the NCI and the University of Colorado. These include activation of the steroidogenic pathway (Figs. 2A and 4), common mutations such as TP53 and β-catenin (Fig. 6), and overexpression of IGF2 (Supplementary Fig. S2B). Moreover, it has recently been suggested for MUC-1 that gene expression cluster type (C1B) and specific mutations (e.g., TP53) are retained from patients to cell lines (https://www.biorxiv.org/content/10.1101/2023.04.05.535576v1). The cell lines also retain features of the PDXs from which they were derived (Fig. 3). Because cell lines are important as models, it is meaningful that the reproducibility and stability of the ACC cell lines was demonstrated in the present study.

Our study reveals that the cell lines also share many genomic signatures with ACC surgical samples, reinforcing their validity as research models. Despite those similarities, some degree of heterogeneity was observed between the ACC cell lines and the ACC surgical samples with respect to steroidogenesis (Fig. 2A), ABC transporter expression (Fig. 2B), *TERT* expression (Fig. 5), *MGMT* expression, MMR status (Fig. 8), and SLFN11 expression (Supplementary Fig. S4). Previous genomics studies on ACC have revealed that differences in the Wnt/β-catenin pathway, p53/Rb pathway, cell-cycle regulation, histone modifications, DNA methylation, steroidogenesis, and immunobiology lead to differences in ACC biological behavior (6, 7, 57). The heterogeneity among samples demonstrated in our study illustrates the complexity and diversity of ACCs and underscores the importance of considering this heterogeneity for classifying ACCs in future studies with the aim of personalized therapeutic approaches.

Adrenocortical differentiation and steroidogenicity of ACCs have been associated with prognosis. Most high ADS cases were grouped in the Clusters of Group III (CoC III), a cluster of poor prognosis cases among ACCs (7, 57). In our ADS analyses, the cell lines and corresponding PDXs are in two groups: one comprising CU-ACC1 and NCI-H295R, a steroid-producing cell line with mutations in β-catenin, and the other containing CU-ACC2, a non–steroid-producing cell line (and the small cell carcinoma cell line SW-13; Supplementary Figs. S2A and S4A). We show here that SW-13 lacks expression of steroid metabolism genes (*SULT2A1* and *CYP11A1*), drug efflux pump (*ABCB1*), and a transcription factor gene involved in adrenocortical cell differentiation (*NR5A1*; Fig. 2A and B; Supplementary Fig. S2A). SW-13 is included in the Sanger/Massachusetts General Hospital Genomics of Drug Sensitivity in Cancer (GDSC) dataset of CellMiner Cross-Database (26), which confirms similar observations (see https://discover.nci.nih.gov/). The consistency of these results contributes to the argument that SW-13 should not be considered an ACC cell line.

Analyses using the ADS and APM scores show that the APM is suppressed in ACCs that differentiate into the adrenocortex and express the steroidogenic pathway (Fig. 4B). This observation is consistent with the view that expression of the steroidogenic pathway in ACC may inhibit immune responses and reduce the efficacy of ICI therapy (58). Notably, the patient from whom the CU-ACC2 cell line was developed, and which has MMR defect and high APM (Fig. 4B), was responsive to ICIs (ref. 59).

Unexpectedly, we found that two of the four cell lines (CU-ACC2 and NCI-H295R) and three out of the six surgical samples lack telomerase reverse transcriptase (*TERT*) expression and that the two cell lines show an ALT phenotype based on PML and TERF2 staining (Fig. 5A and B). Our study did not detect the local amplification of TERT and TERF2 genes reported in 15% and 7% of ACC cases, respectively (7), and this difference may be due to our limited sample size. However, our findings of absent TERT expression in a significant proportion of samples displaying the ALT phenotype align with recent studies showing that a subset of ACCs exhibit the ALT phenotype and have a poor prognosis (27, 60), underscoring the relevance of our results. The ALT phenotype is also strongly associated with loss of ATRX or DAXX; ATRX and DAXX together form a complex that deposits the noncanonical histone variant H3.3 in pericentromers and telomeric heterochromatin (61–63). ATRX/DAXX mutations are often truncating nonsense mutations, and they are often observed in ACC cases (7). MUC-1 has homozygous deleterious mutations in ATRX, and JIL-2266 has mutations in DAXX (Fig. 5C). Thus, these cell lines may exhibit ALT phenotype. Further studies are warranted to further explore telomere maintenance in ACC (60).

Finally, ACC_CellMinerCDB allows the exploration of therapeutically relevant features and opportunities and biomarkers for ACCs. We find that expression of *SOAT1*, the target of mitotane (47), predicts the activity of mitotane determined both in our laboratory and at the NCATS (Fig. 7). This could be important as only a fraction of patients respond to mitotane and mitotane is often poorly tolerated. Marked overexpression of the drug efflux transporter MDR-1 was observed in the ACC cell lines and surgical samples (Fig. 2B; Supplementary Fig. S2). This overexpression was associated with resistance to specific chemotherapies in the ACC cell lines, suggesting the importance of assessing ABC transporter expression by RNA-seq in ACC patient samples, in which case alternative therapeutic strategies should be considered (Supplementary Fig. S1). Our findings highlight the possibility of repurposing temozolomide, a drug commonly used to treat brain tumors, for ACC therapy when MGMT is absent in tumor samples while the cancer cells maintain proficient mismatch repair to kill them (Fig. 8; ref. 64). SLFN11 expression was also suggested to be a promising marker for ACC therapy. SLFN11 is associated with responses to DNA-damaging agents (65), and its expression in ACC surgical specimens in the present study supports the possibility that SLFN11 may serve as a marker to predict ACC patient responsiveness to specific treatments (Supplementary Fig. S4).

We recognize the limitations of this study, especially the small number of cell lines included and the fact that one of the cell lines, SW-13, was not identified as ACC. These limitations highlight the need to further develop a larger cohort of ACC preclinical models including cell lines, organoids, and additional PDXs. Nevertheless, we anticipate that collaborative efforts in generating the state-of-the-art ACC_CellMinerCDB, the first and only genomic database designed specifically for ACC preclinical models, will expand our knowledge of ACC biology and novel therapeutic targets, providing a foundation for more personalized treatment strategies for patients.

## Supplementary Material

Figure S1 Supplement Figure 1 related to Figure 2

Figure S2 Supplement Figure 2 related to Figure 4

Figure S3 Supplement Figure 3 related to Figure 8

Figure S4 Supplement Figure 4

Table S1 Supplement Table 1

Table S2 Supplement Table 2, related to Figure 5 and 6.
