# Supplementary material for: A Database Tool Integrating Genomic and Pharmacologic Data from Adrenocortical Carcinoma Cell Lines, PDX, and Patient Samples
Source: Cancer Res Commun. 2024 Sep 11;4(9):2384–98. doi: 10.1158/2767-9764.CRC-24-0100 (PMC11389377; doi:10.1158/2767-9764.CRC-24-0100)
Supplement: Figure S1 — Supplement Figure 1 related to Figure 2 [file crc-24-0100_figure_s1_supps1.pdf]

Supplement Figure 1 related to Figure 2.

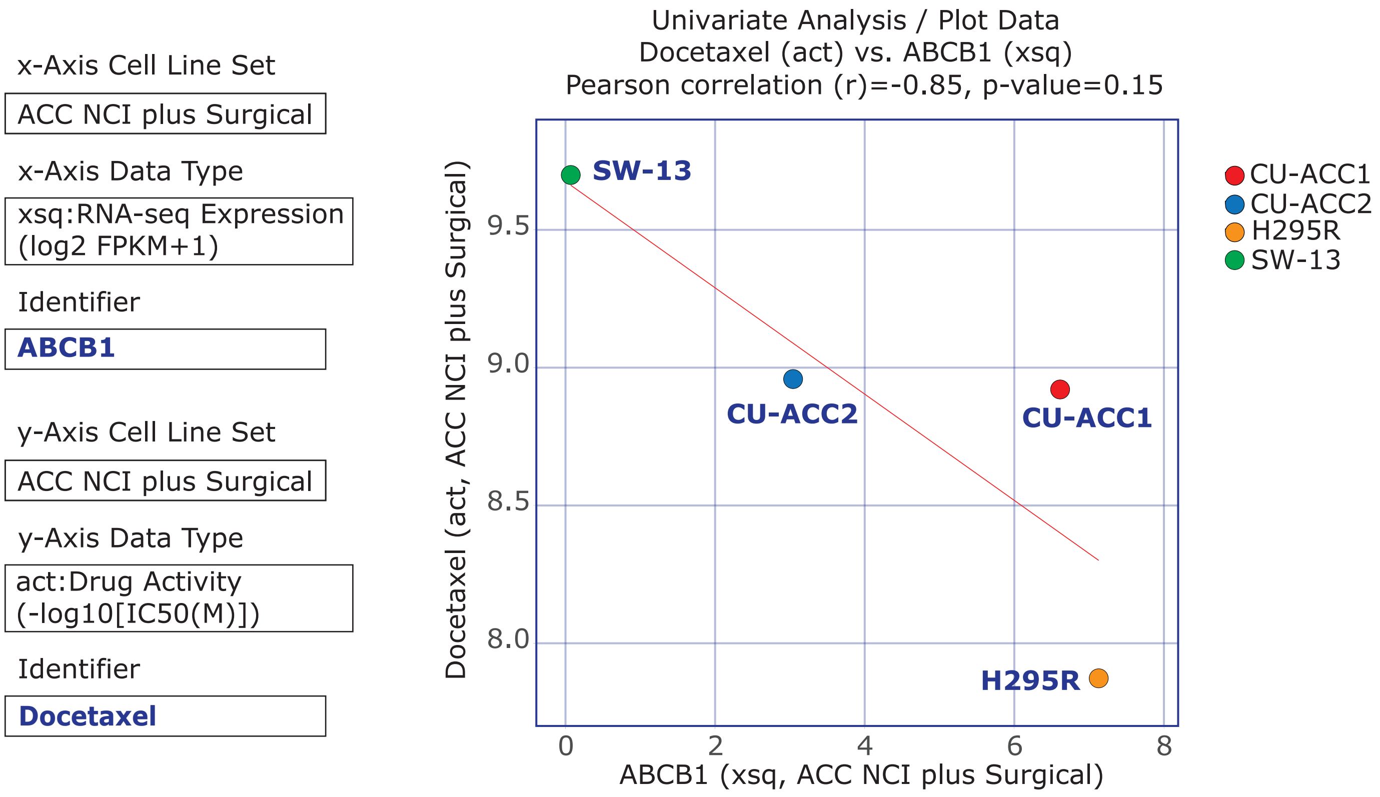

Overexpression of ABCB1 (MDR-1) is correlated with resistance to docetaxel.
