# Supplementary material for: A Database Tool Integrating Genomic and Pharmacologic Data from Adrenocortical Carcinoma Cell Lines, PDX, and Patient Samples
Source: Cancer Res Commun. 2024 Sep 11;4(9):2384–98. doi: 10.1158/2767-9764.CRC-24-0100 (PMC11389377; doi:10.1158/2767-9764.CRC-24-0100)
Supplement: Figure S2 — Supplement Figure 2 related to Figure 4 [file crc-24-0100_figure_s2_supps2.pdf]

Supplement Figure 2 related to Figure 4.

A

x-Axis Cell Line Set

ACC NCI plus Surgical

x-Axis Data Type

mda:Signatures, Miscellaneous data

Identifier

ADS

y-Axis Cell Line Set

ACC NCI plus Surgical

y-Axis Data Type

xsq:RNA-seq Expression (log2 FPKM+1)

Identifier

NR5A1

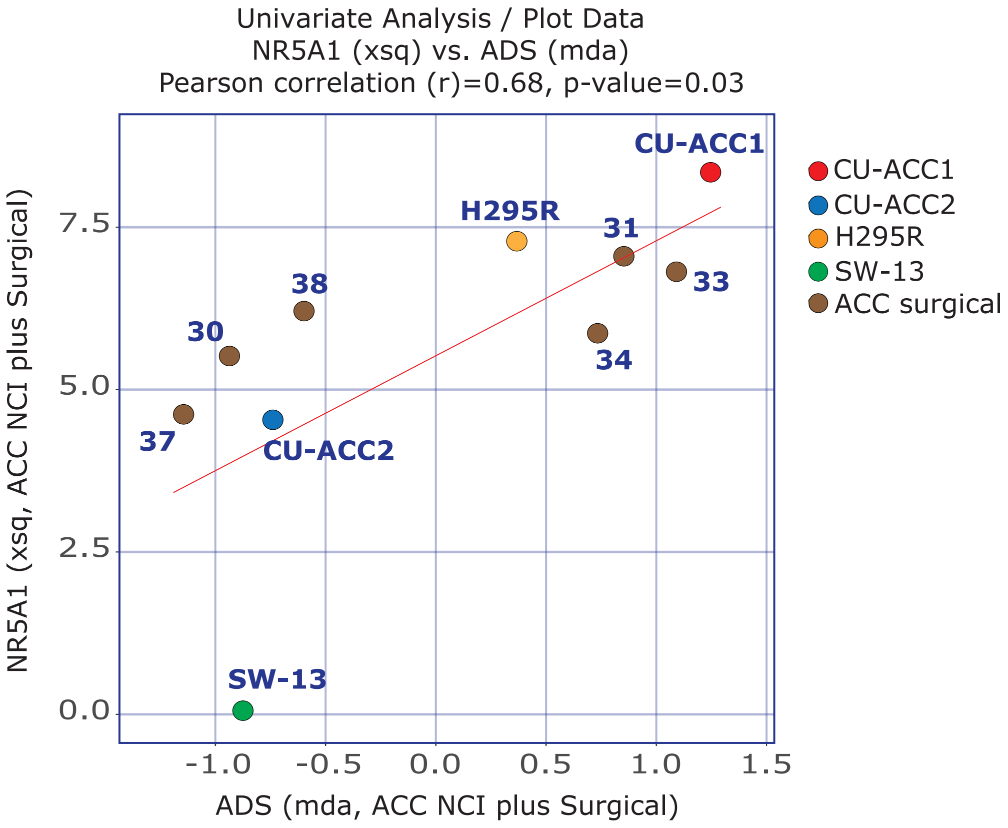

B

x-Axis Cell Line Set

ACC NCI plus Surgical

x-Axis Data Type

xsq:RNA-seq Expression (log2 FPKM+1)

Identifier

DLK1

y-Axis Cell Line Set

ACC NCI plus Surgical

y-Axis Data Type

xsq:RNA-seq Expression (log2 FPKM+1)

Identifier

IGF2

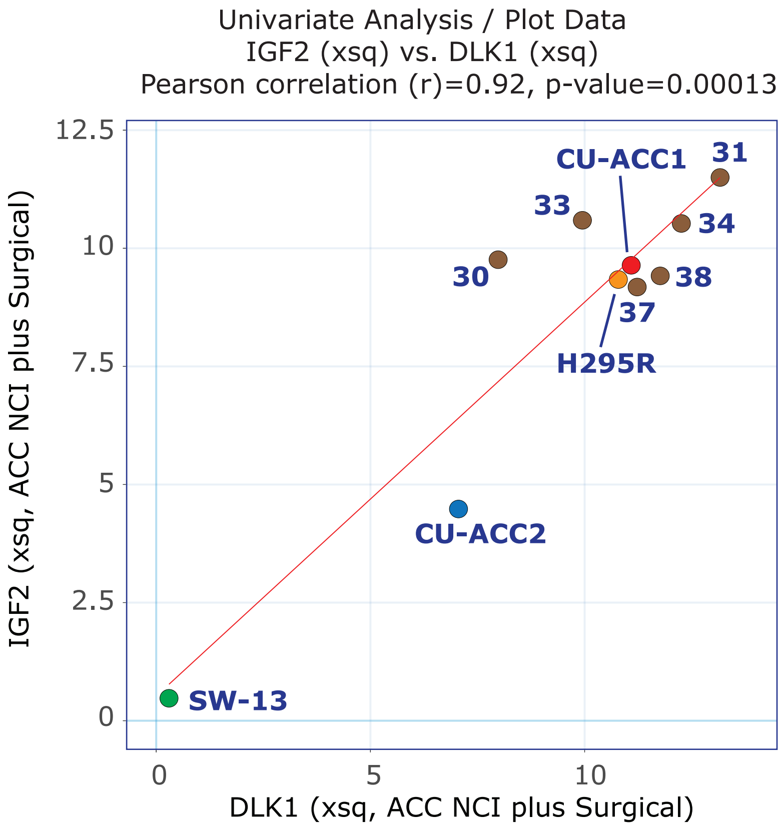

(A) Correlation between the ADS score and the Nuclear Receptor Subfamily 5, Group A, Member 1 NRSA1, which also referred to as Steroidogenic Factor-1 (SF1).

(B) Overexpression of IGF2 in ACC cell lines and tumor samples and correlation with the expression of the surface receptor gene DLK1.
