# Supplementary material for: A Database Tool Integrating Genomic and Pharmacologic Data from Adrenocortical Carcinoma Cell Lines, PDX, and Patient Samples
Source: Cancer Res Commun. 2024 Sep 11;4(9):2384–98. doi: 10.1158/2767-9764.CRC-24-0100 (PMC11389377; doi:10.1158/2767-9764.CRC-24-0100)
Supplement: Figure S3 — Supplement Figure 3 related to Figure 8 [file crc-24-0100_figure_s3_supps3.pdf]

Supplement Figure 3 related to Figure 8

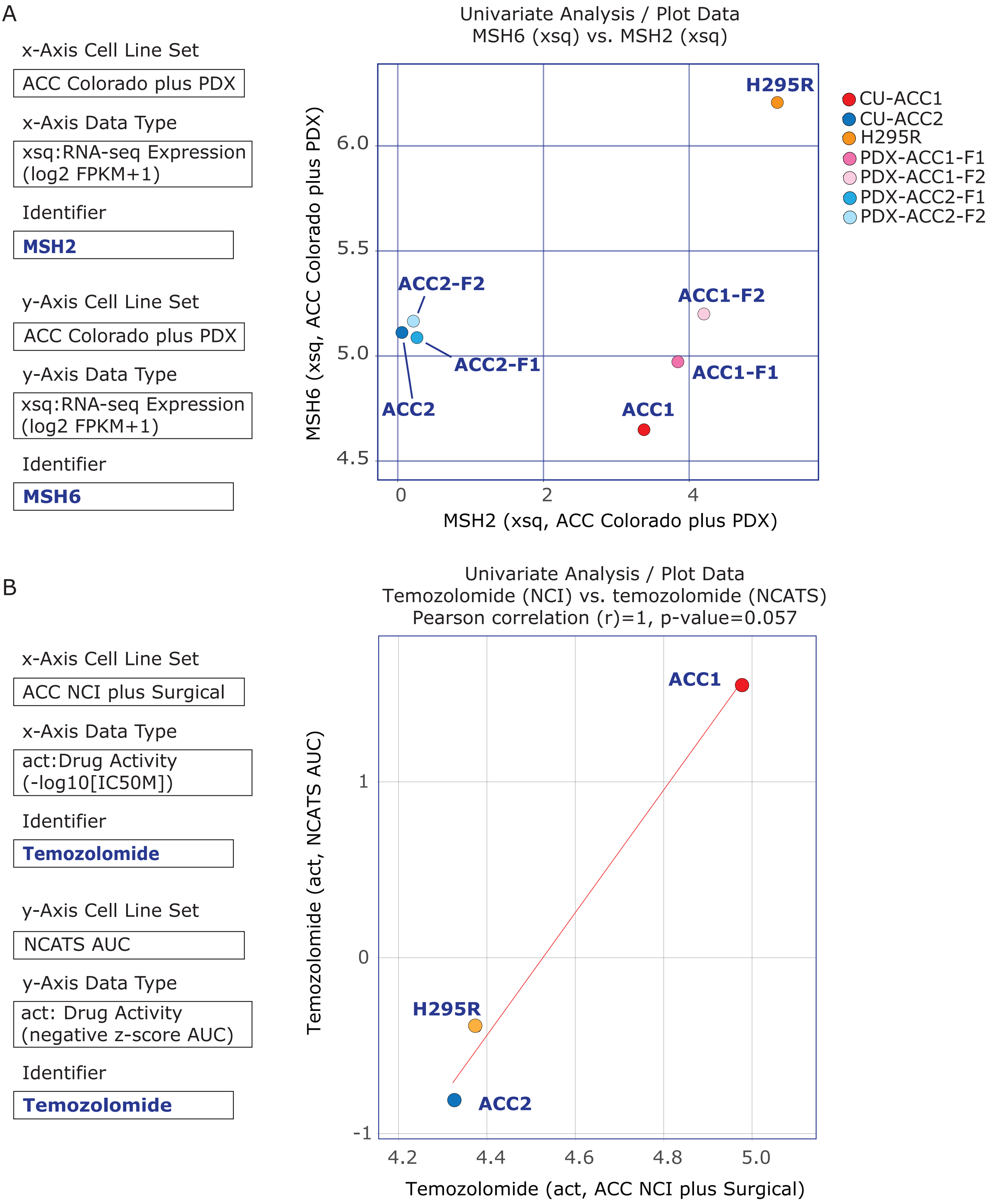

(A) Mismatch repair deficiencies in the ACC2 cell line and its related PDXs.
(B) Sensitivity and resistance of CU-ACC1 and CU-ACC2 and NCI-H295R to temozolomide both in the NCI and NCATS databases. Resistance of ACC2 is related to lack of MSH2 expression in CU-ACC2 (see Figure 8B).
