# Supplementary material for: A Database Tool Integrating Genomic and Pharmacologic Data from Adrenocortical Carcinoma Cell Lines, PDX, and Patient Samples
Source: Cancer Res Commun. 2024 Sep 11;4(9):2384–98. doi: 10.1158/2767-9764.CRC-24-0100 (PMC11389377; doi:10.1158/2767-9764.CRC-24-0100)
Supplement: Figure S4 — Supplement Figure 4 [file crc-24-0100_figure_s4_supps4.pdf]

Supplement Figure 4

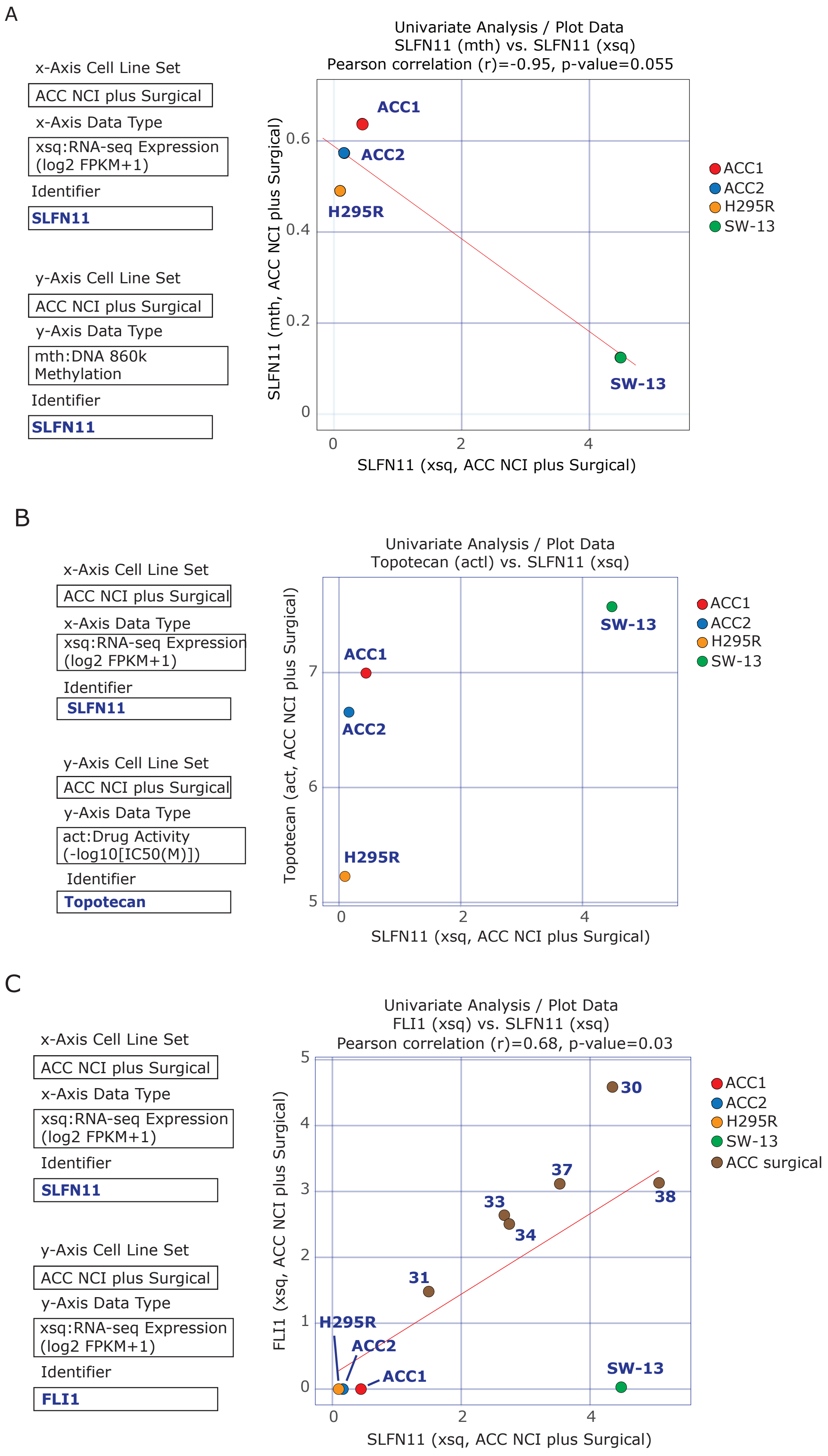

(A) Lack of SLFN11 expression in the ACC cell lines CU-ACC1, CU-ACC2 and NCI-H295R is related to promoter hypermethylation.
(B) SW-13 cells, which express SLFN11 are the most sensitive to topotecan.
(C) Lack of SLFN11 expression and of its transcription factor FLI1 in the ACC cell lines and correlation between SLFN11 and FLI1 expression in the ACC surgical samples.
